# Stress–Strain Behavior of Crushed Concrete as a Special Anthropogenic Soil

**DOI:** 10.3390/ma16237381

**Published:** 2023-11-27

**Authors:** Katarzyna Gabryś, Katarzyna Dołżyk-Szypcio, Zenon Szypcio, Wojciech Sas

**Affiliations:** 1 Water Centre WULS, Warsaw University of Life Sciences—WULS, 6 Ciszewskiego Street, 02-787 Warsaw, Poland; wojciech_sas@sggw.edu.pl; 2 Department of Geotechnics, Roads and Geodesy, Faculty of Civil Engineering and Environmental Sciences, Bialystok University of Technology, 45A Wiejska Street, 15-351 Bialystok, Poland; z.szypcio@pb.edu.pl

**Keywords:** crushed concrete, triaxial compression tests, stress–dilatancy, frictional state concept

## Abstract

The stress–plastic dilatancy relationship was investigated for crushed concrete during drained and undrained triaxial compression tests in the light of the frictional state concept. The slope of the dilatant failure state line is greater than that of quartz sand for drained triaxial compression due to the crushing effect. The crushing effect parameters for drained and undrained conditions are very similar. Due to the very angular shape of crushed concrete grains, the crushing effect is observed at low stress levels. Some characteristic behaviors of geomaterials during shear are visible only in the stress ratio–plastic dilatancy plane and are very rarely presented in the literature. The stress ratio–plastic dilatancy relationship, which is basic in elastic–plastic modeling of geomaterials, can be described using the frictional state concept.

## 1. Introduction

Industrial and human activities generate rapidly increasing amounts of waste, such as crushed concrete, broken glass, plastics, used tires, and others. With rapid urbanization, new structures are replacing old ones, and renovations and demolitions of existing buildings produce large amounts of concrete waste [1]. The growing demand for aggregates for the construction of engineering structures has begun to pose a threat to available natural resources. Natural aggregates are gradually being exhausted around the world. The use of recycled materials in construction is very beneficial from an economic and ecological point of view [2,3,4]. Over the last 15 years, there has been extensive research into the recycling of demolition waste to replace natural aggregates (NAs). As a result, recycled materials have emerged as an alternative to NAs [5,6]. Recycled aggregate (RA) was first used in road construction during the Second World War in England [7]. For decades, scientists have been working intensively on using various types of waste in various engineering projects. 

The subject of this study is recycled concrete aggregate (RCA). This concept includes aggregates obtained by recycling clean concrete waste where the content of other building waste must be very low—below a few percent [8,9]. The RCA characterization indicates a lower density and unit weight, as well as a higher crushing value and water absorption, compared with natural aggregate [10]. Recycled concrete aggregates (RCAs) are often used to produce new concrete. Concrete with the addition of RCA has a higher mortar content than the initial concrete and a greater water absorption capacity [11]. Increasing the amount of RCA in the concrete mix reduces the compressive strength and elasticity modulus of the concrete [10]. RCA is more reliable if the properties are homogeneous and meet the required standards [12]. RCA has recently gained popularity as a valuable raw material and is widely used in pavement construction [13] and embankments, and as a ground improvement material [14]. The addition of RCA, glass aggregates, and tire-derived aggregates to the soil can significantly improve its resistance to liquefaction [15]. The increasing demands on modern geotechnical structures require knowledge of many mechanical parameters of waste materials and soil mixtures subjected to monotonic, cyclical, and dynamic loads [16,17,18,19,20,21].

In elastoplastic modeling, the stress–plastic dilatancy relationship plays a key role [22,23]. In many geomaterial models, a critical state is taken as the basis. Recently, a new frictional state concept (FSC) was presented for geomaterials under triaxial compression [24] as an extension of the critical state concept [25]. In this work, the stress–plastic dilatancy relationships for drained and undrained triaxial compression tests were investigated in the light of the FSC.

## 2. Stress–Plastic Dilatancy Relationship for Soils

The general stress ratio–plastic dilatancy relationship using the frictional state concept (FSC) is as follows [24]:

(1) $$\eta =Q-AD^{p},$$

where

(2) $$\eta =q/p^{\prime },$$

(3) $$Q=M^{o}-\alpha A^{o},$$

(4) $$A=\beta A^{o},$$

(5) $$D^{p}={\delta \varepsilon }_{\upsilon }^{p}/{\delta \varepsilon }_{q}^{p},$$

(6) $$A^{o}=1-M^{o}\left(\delta q/\delta p^{\prime }\right),$$

(7) $$q=\sigma_{1}^{\prime }-\sigma_{3}^{\prime },$$

(8) $$p^{\prime }=\left(\sigma_{1}^{\prime }+2\sigma_{3}^{\prime }\right)/3,$$

$\sigma_{1}^{\prime }=\sigma_{a}^{\prime }$, $\sigma_{3}^{\prime }=\sigma_{c}=p_{0}$ are the confining pressure constant during shear; *α* and *β* are the new soil parameters of the FSC. The plastic parts of the volumetric and shear strain increments are:

(9) $${\delta \varepsilon }_{\upsilon }^{p}={\delta \varepsilon }_{\upsilon }-{\delta \varepsilon }_{\upsilon }^{e},$$

(10) $${\delta \varepsilon }_{q}^{p}={\delta \varepsilon }_{q}-{\delta \varepsilon }_{q}^{e},$$

(11) $${\delta \varepsilon }_{\upsilon }={\delta \varepsilon }_{1}+2{\delta \varepsilon }_{3},$$

(12) $${\delta \varepsilon }_{q}=2\left({\delta \varepsilon }_{1}-{\delta \varepsilon }_{3}\right)/3,$$

where

(13) $${\delta \varepsilon }_{\upsilon }^{e}=\delta p^{\prime }/K,$$

(14) $${\delta \varepsilon }_{q}^{e}=\delta q/3G,$$

$\varepsilon_{1}=\varepsilon_{a}$, $\varepsilon_{3}=\varepsilon_{a}$; *K* and *G* are the elastic bulk and shear modulus, respectively. Poisson’s ratio can be calculated using

(15) $$\nu =\frac{3K-2G}{2\left(3K+G\right)}.$$

For triaxial compression ($\sigma_{2}^{\prime }=\sigma_{3}^{\prime }$),

(16) $$M=M_{c}^{o}=6\sin\phi^{o}/\left(3-\sin\phi^{o}\right),$$

where $\phi^{o}$ is the angle of friction at the critical frictional state [24,25]. The definition of critical frictional state and the calculation procedure using $\phi^{o}$ were provided by Szypcio [24]. For conventional drained triaxial compression, $\delta q/\delta p^{\prime }=3,$ and

(17) $$A=A_{c}^{o}=1-\left(M_{c}^{o}/3\right).$$

For undrained triaxial compression, the stress path [25] is undefined. The correct stress ratio–plastic dilatancy relationship [25,26] is obtained for

(18) $$A=A_{c}^{o}=1+\left(2M_{c}^{o}/3\right).$$

**Figure 1:**
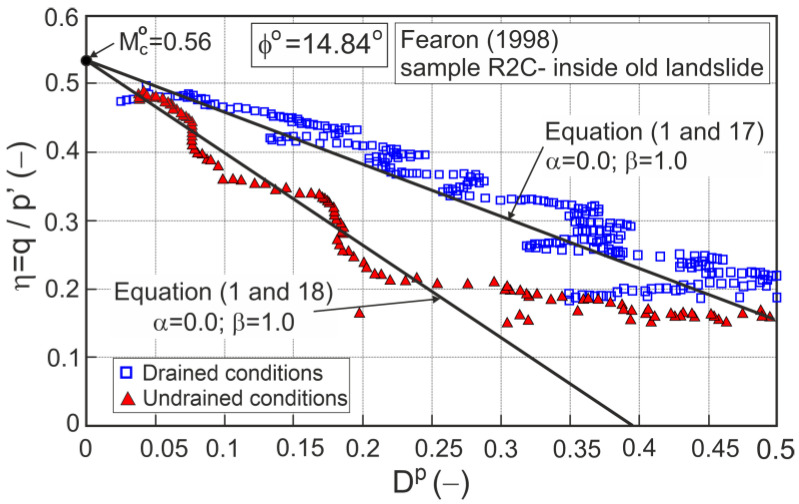
Stress ratio–plastic dilatancy relationships for clay taken from the Acquara-Vadoncello old landslide (adopted from [27]).

An example of correct stress ratio–plastic dilatancy relationships for drained and undrained conditions for fully destructured clay taken from the Acquara-Vadoncello old landslide [27] is shown in Figure 1.

## 3. Test Material

**Figure 2:**
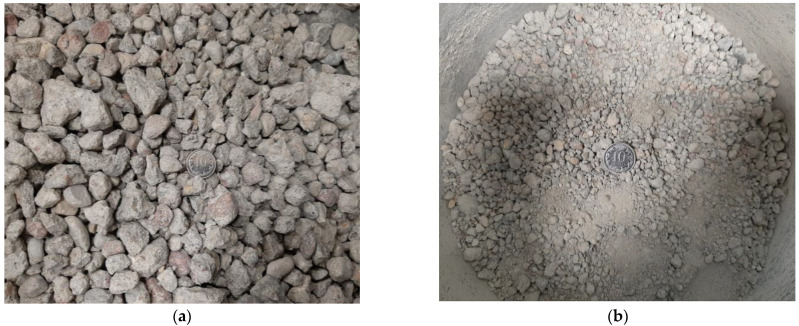
View of tested RCA: (**a**) raw crushed concrete; (**b**) prepared final mixture.

In this study, recycled concrete aggregate (RCA) from the demolition waste material from the construction of concrete façade walls of Warsaw buildings in the 1990s was used. The material was supplied as damaged concrete cubic samples with dimensions of 150 × 150 × 150 mm. The strength class of the analyzed RCA was determined to be between C16/20 and C30/37 according to [28]. The raw material was crushed through a five-stage Proctor crushing and fractionated using a sieve separation process. The resulting mixtures were used for further laboratory testing. The current paper presents only the results for one selected blend, i.e., fraction 0–7 mm. The images of the test material before crushing and after the mixing process to the final blend are provided in Figure 2. Before being crushed, the grains were sharp-edged and came in various shapes, blades, discs, or cylinders, according to Zingg’s classification [29]. After crushing, the shape of the grains became more uniform and spherical.

**Figure 3:**
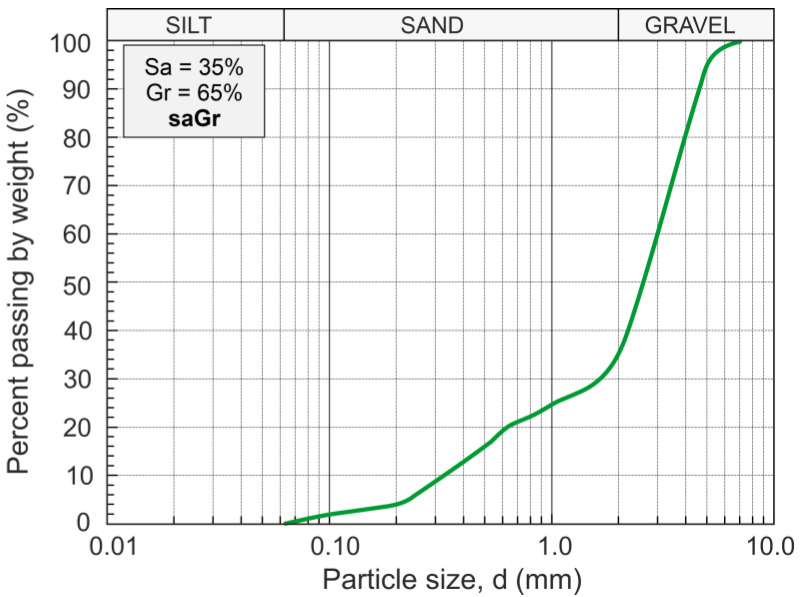
Particle size distribution of examined RCA.

For the investigated RCA material, the dominant component was broken cement concrete (around 99%), and less than 1% was glass and brick. During the preparation of the target mixes, the glass and rock elements were removed. Conventional laboratory tests and procedures typical for natural soils were used in the geotechnical characterization of all prepared concrete aggregate specimens. In Figure 3, the distribution curve of the particle size of the RCA employed in the tests is shown. The grain size distribution analysis was performed according to [30]. This analysis led to the classification of the tested anthropogenic material as gravel with sand (saGr), according to [31]. 

The selected physical properties of RCA and leachate concentration from the RCA mixture are presented in Table 1 and Table 2. In Table 1, the typical characteristics for natural soils are included, like the values of the mean diameter (d_50_) and the coefficients (C_u_, C_c_) or parameters that characterize the process of compaction. Note: These are average values, characterizing the entire test material. The obtained grain-size indicators classified the RCA employed in the tests as a well-graded material, susceptible to compaction, and suitable for earthworks. The minimum dry density (ρ_d,min_) is the result of vibration testing [32]. The optimum moisture content (OMC) and maximum dry density (ρ_d,max_) were estimated using the Proctor method. According to Sulewska [33], in the case of RCA, it is recommended to use the Proctor method for compaction, although it is characterized by a grain size indicative of non-cohesive soil. From the compactibility results, it can be concluded that the tested RCA compacted similarly to natural aggregates, where a characteristic value of optimum moisture content was observed. The phenomenon of the strong correlation of OMC with ρ_d,max_, and with the narrow moisture range observed in this study, points to coarse soils with low fine fractions. This phenomenon resembles the compaction of gravel with a low clay content [34]. 

The methodology for preparing samples and aqueous extracts for the determination of water-soluble chloride and sulfate concentrations was based on the standard [35]. Sulfates and dissolved chlorides were determined based on the methodology given by Kiedryńska et al. [36]. For heavy metals, the methodology was taken from the standard [37]. The concentration of heavy metals in the aqueous extracts was measured using atomic absorption spectrometry (type of atomization: flame). In addition, the electrolytic conductivity and pH of the aqueous extracts prepared from the RCA were tested. The results obtained indicate that the leachability of chlorides, sulfates, and selected heavy metals from the tested concrete aggregate was below the permissible limits. In conclusion, the RCA investigated in the laboratory was chemically safe for the soil and water environment when used, for example, in unbound mixtures for road pavement foundations or water damming embankments.

## 4. Test Procedure

**Figure 4:**
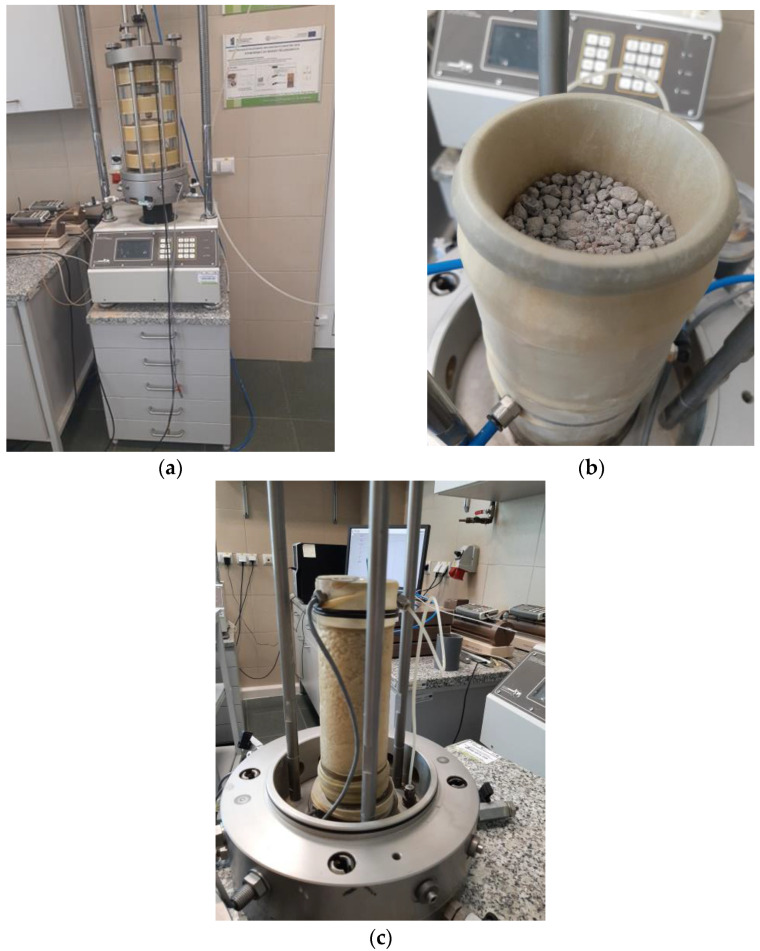
View of: (**a**) triaxial test apparatus; (**b**) specimen during molding; (**c**) prepared final mixture.

An experimental investigation program was carried out in order to gain insight into the stress–strain behavior of the studied RCA mixtures. A total of nine specimens (denoted by the symbols RCA-1 to RCA-9) were tested employing standard monotonic triaxial tests, according to the standard [38]. The automated triaxial testing system (Figure 4a) adopted for this study is described in [39,40]. The tests were carried out on remolded samples prepared either in a loose state (dry tamping method; specimens RCA-5 to RCA-9) or in a compacted state (moist tamping method; specimens RCA-1 to RCA-4) based on OMC and ρ_d,max_. All the specimens had a height of about 140 mm and a diameter of approx. 70 mm, and they were prepared in a special triplicate mold located on the base of the apparatus. In Figure 4b, there is an exemplary illustration of the triaxial specimen during formation; Figure 4c shows the final mixture.

Each specimen was saturated and initially flushed with de-aerated water. Thereafter, a high back pressure (minimum 240 kPa and more) was applied. The Skempton parameter B values obtained for RCA mixtures were B = 0.97–0.99. When the saturation was complete (back pressure method [41]), the specimens were isotropically consolidated. The monotonic triaxial tests were executed in two ways, either in drained conditions (CD) (for specimens RCA-1 to RCA-6) or in undrained conditions (CU) (for specimens RCA-7 to RCA-9), according to the assumed testing procedure (Table 3). Specimen height changes, shear force value, and eventually pore water pressures were recorded during shearing. For the results of this experimental study to be applicable in engineering practice, a range of confining stresses from 45 kPa to 400 kPa [16] were utilized in the tests (*p′* = 45 kPa for RCA-1, *p′* = 90 kPa for RCA-2, *p′* = 180 kPa for RCA-3, *p′* = 270 kPa for RCA-4, *p′* = 50 kPa for RCA-7, *p′* = 200 kPa for RCA-5 and RCA-8, *p′* = 400 kPa for RCA-6 and RCA-9). The shear rate of each specimen was set at 0.033 mm/min based on the oedometric tests performed and the experience of the researchers [42]. In addition, at the end of the consolidation process of some mixtures, the small-strain stiffness ($G_{0}$) was determined using the bender element (BE) test method. The BE test involves the use of a pair of piezoelectric ceramic transducers to transmit or receive a mechanical disturbance, from which the measurement of the shear wave velocity (Vs) can be made. Such disturbance is generated by a voltage signal in the BE transmitter and detected by the BE receiver that converts it into another voltage signal [43,44,45]. The piezoelectric transducers for BE testing in the triaxial chamber were used. The values of the estimated $G_{0}$ modulus for the selected test mixtures are summarized in Table 3. 

## 5. Methodology

**Figure 5:**
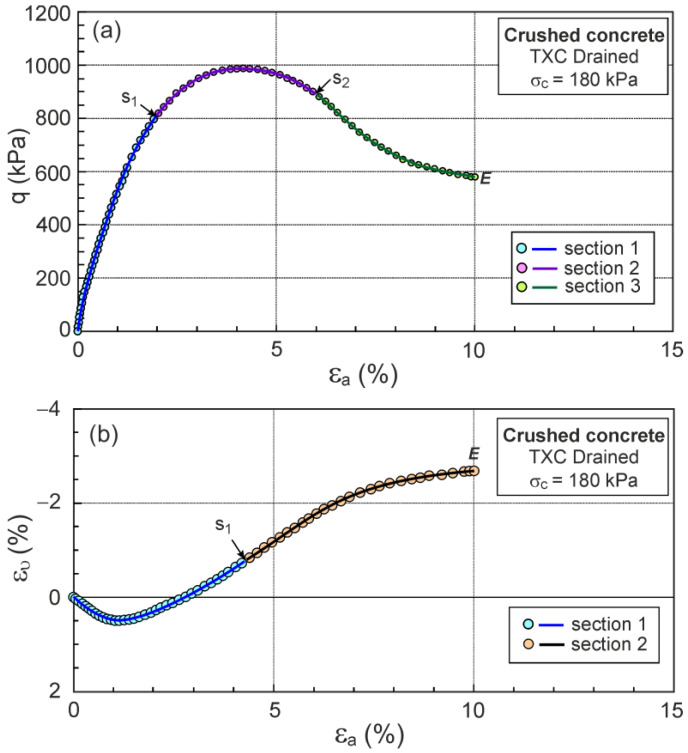
Example of approximations of experimental values of $\sigma_{1}^{\prime }/\sigma_{3}^{\prime }$ and $\varepsilon_{\upsilon }$: (**a**) $\sigma_{1}^{\prime }/\sigma_{3}^{\prime }-\varepsilon_{1}$; (**b**) $\varepsilon_{\upsilon }-\varepsilon_{1}$.

The experimental values of q and $\varepsilon_{\upsilon }$ as functions of $\varepsilon_{1}\left(\varepsilon_{a}\right)$ were approximated sectionally using high-degree polynomials (Figure 5).

Attention was focused on achieving the continuity of the approximate values but also their increments at the section connection points. Other stress and strain values were calculated using the following equations:

(19) $$\sigma_{1}^{\prime }/\sigma_{3}^{\prime }=\left(q/\sigma_{3}^{\prime }\right)+1,$$

(20) $$p^{\prime }=\frac{1}{3}\sigma_{3}^{\prime }\left(\sigma_{1}^{\prime }/\sigma_{3}^{\prime }+2\right),$$

(21) $$\eta =q/p^{\prime },$$

(22) $$\varepsilon_{q}=\varepsilon_{1}-\frac{1}{3}\varepsilon_{\upsilon },$$

(23) $$D={\delta \varepsilon }_{\upsilon }/{\delta \varepsilon }_{q},$$

**Figure 6:**
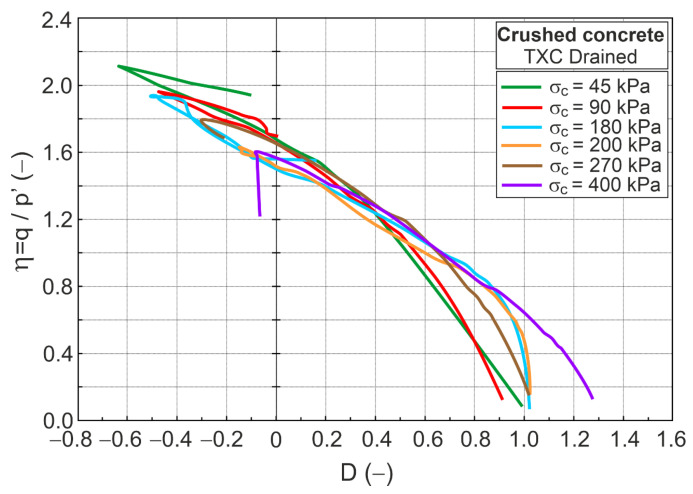
The stress ratio–dilatancy relationship for drained tests.

The stress ratio–dilatancy relationship for the experimental test values is shown in Figure 6.

## 6. Elasticity Parameters

To be able to analyze the relationship between stress ratio and plastic dilatancy ($\eta -D^{p}$), it is necessary to know the elasticity parameters. Assuming the elastic behavior of soil at the beginning of shear, the shear and bulk modulus can be calculated using:

(24) $$G^{*}=\frac{1}{3}{\left(\frac{\delta q}{\delta \varepsilon_{q}}\right)}_{0},$$

(25) $$K^{*}={\left(\frac{\delta p^{\prime }}{\delta \varepsilon_{\upsilon }}\right)}_{0},$$

The values δq, $\delta p^{\prime }$, ${\delta \varepsilon }_{q}$, ${\delta \varepsilon }_{\upsilon }$, $G^{*}$, and $K^{*}$ were calculated for $\varepsilon_{a}={\delta \varepsilon }_{a}=0.01\%$. The calculated values of $G^{*}$ and $K^{*}$ and the experimental values of maximum shear modulus $G_{0}$ are shown in Table 4. 

**Figure 7:**
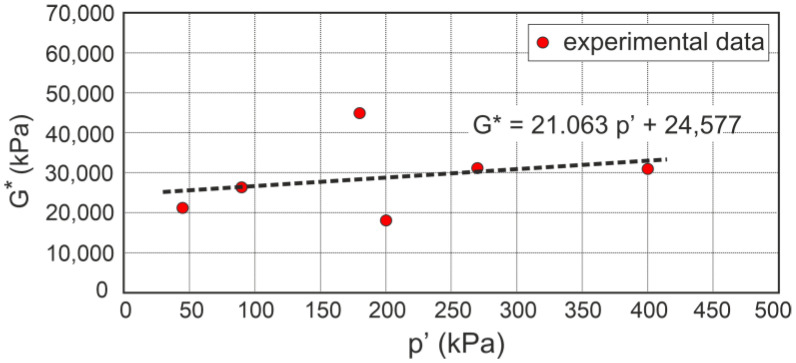
Effect of the mean effective stress on the calculated shear modulus.

The experimental values of $G_{0}$ are higher than the calculated values of $G^{*}$. The difference is influenced by unidentified errors in the measurement of strains at the beginning of shear and approximation errors. In further calculations, it is assumed that

(26) $$G=a_{\mathrm{avg}}\;G^{*},$$

where $a_{avg}=$ 3.758 is the average value of the ratios (Table 4)

(27) $$a=G_{0}/G^{*},$$

and $G^{*}$ is a function of the mean effective stress

(28) $$G^{*}=21.063\;p^{\prime }+24,577,$$

where $p^{\prime }$ and $G^{*}$ are expressed in kPa (Figure 7).

**Figure 8:**
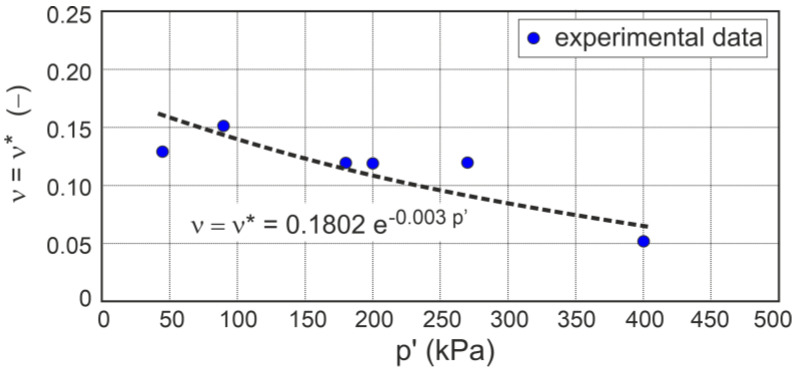
Effect of the mean effective stress on Poisson’s ratio.

It is also assumed that Poisson’s ratio for drained triaxial compression is influenced by the stress level and can be expressed as (Figure 8)

(29) $$\nu =\nu^{*}=0.1812\;e^{-0.003\;p^{\prime }},$$

where $p^{\prime }$ is expressed in kPa.

The bulk modulus (*K*) adapted for further calculations was calculated from

(30) $$K=\frac{2G\left(1+\nu \right)}{3\left(1-2\nu \right)},$$

where G and ν are defined in Equations (26) and (29), respectively.

**Figure 9:**
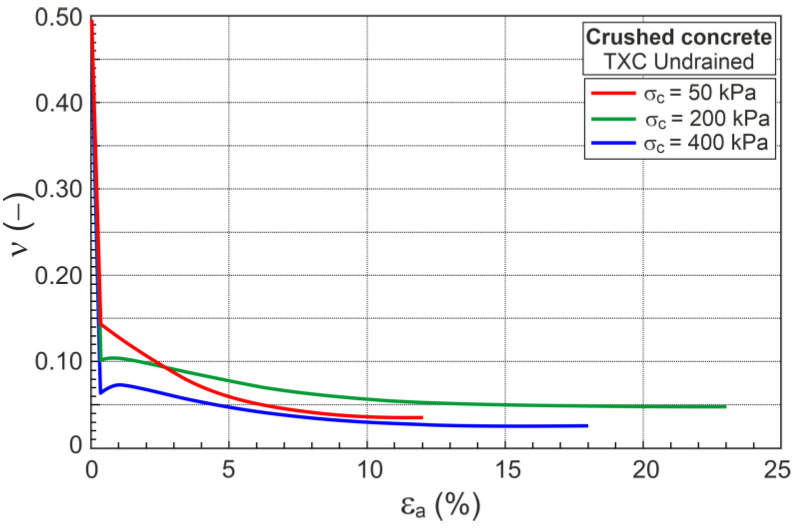
Change in Poisson’s ratio during undrained shear.

For undrained triaxial compression, as for drained conditions, the shear modulus is defined in Equations (26) and (28). The Poison’s ratio of crushed concrete, like Toyoura sand [26], is assumed to change during undrained shear and can be given by

(31) $$\nu =\left\{\begin{array}{ccc} 0.495-\frac{\varepsilon_{a}}{0.35}\left(0.495-\nu_{a}\right) & for & \varepsilon_{a}\leq 0.35\%\\ 0.1802\;e^{-0.003\;p^{\prime }} & for & \varepsilon_{a}>0.35\% \end{array}\right.$$

where $\nu_{a}$ is the value of *ν* calculated from Equation (29) for $p^{\prime }$ at $\varepsilon_{a}=0.35\%$ (Figure 9).

The bulk modulus is calculated using Equation (30), with *G* and *ν* defined in Equations (26) and (31), respectively. 

Knowing the elasticity parameters (*G*, *K*), approximated shear test data (q, $p^{\prime }$, $\varepsilon_{\upsilon }$, $\varepsilon_{q}$), and their increments (δq, $\delta p^{\prime }$, ${\delta \varepsilon }_{\upsilon }$, ${\delta \varepsilon }_{q}$), the plastic parts of volumetric and shear strain increments (${\delta \varepsilon }_{\upsilon }^{p},\;{\delta \varepsilon }_{q}^{p}$) and stress ratio–plastic dilatancy relationship ($\eta -D^{p}$) can be calculated.

## 7. Stress Ratio–Plastic Dilatancy Relationship

### 7.1. Drained Conditions

**Figure 10:**
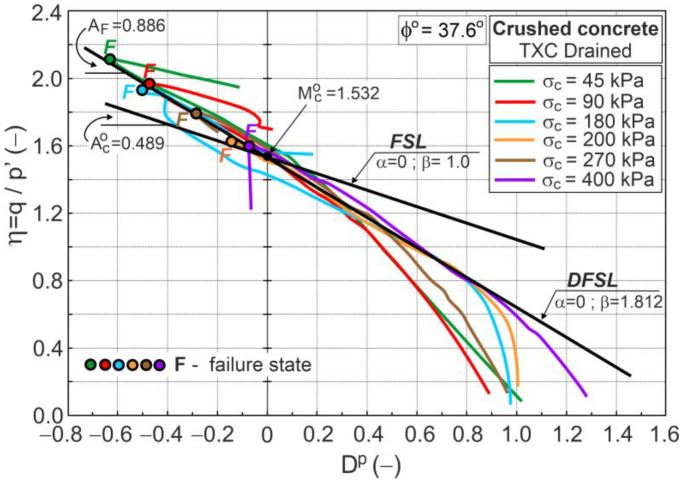
Stress ratio–plastic dilatancy relationships for drained triaxial compression.

Figure 10 shows the stress ratio–plastic dilatancy relationship for the drained triaxial compression test of crushed concrete.

The dilatant failure states (DFSs) and maximum curvature of $\eta -D^{p}$ relationships [24] can be easily identified. Points F in Figure 10 represent the DFSs. A straight line approximating the DFSs, called the dilatant failure state line (DFSL) defined by Equation (1), intersects the vertical axis at η=Q=1.532, and its slope is $A=A_{F}=0.886$ (Figure 10). For granular material, $Q=M_{c}^{o}=1.532$ [22], so the critical frictional state angle $\phi^{o}=37.6{}^{\circ}$. For conventional drained triaxial compression, $A_{c}^{o}=0.489$ (Equation (17)); hence, $\beta =\beta_{F}=A_{F}/A_{c}^{o}=1.812$. The value of $\beta_{F}>1.0$ ($A_{F}>A_{c}^{o}$) represents the intensity of grain crushing during shearing [22]. Because crushed concrete grains have a very angular shape, the crushing effect occurs at low stress levels, similar to limestone gravel and railway ballast [46,47,48,49].

**Figure 11:**
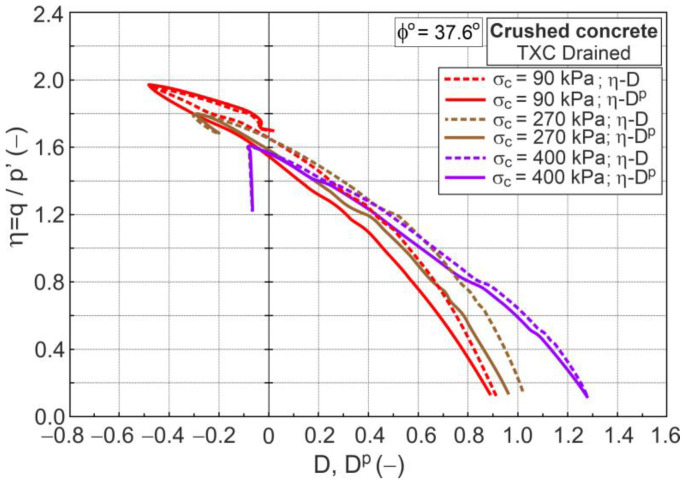
The η−D and $\eta -D^{p}$ relationships for drained triaxial compression.

The relationships η−D and $\eta -D^{p}$ are shown in Figure 11.

Differences are observed not only in the initial but also in the advanced shear stages.

**Figure 12:**
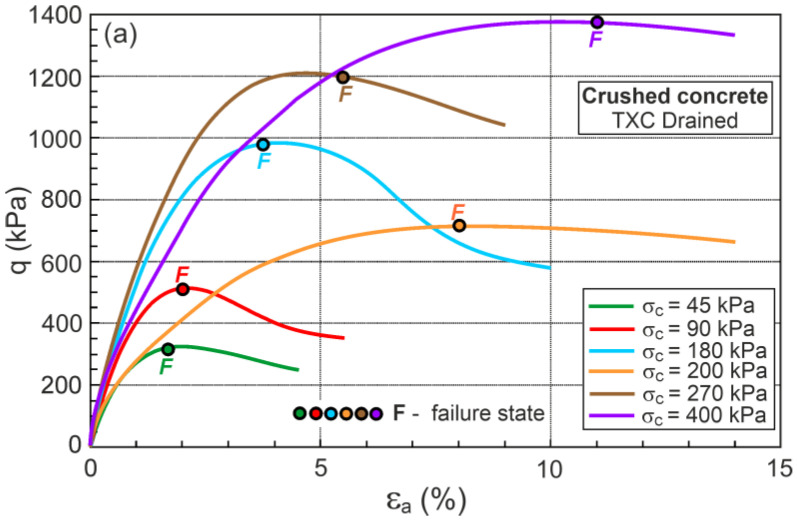

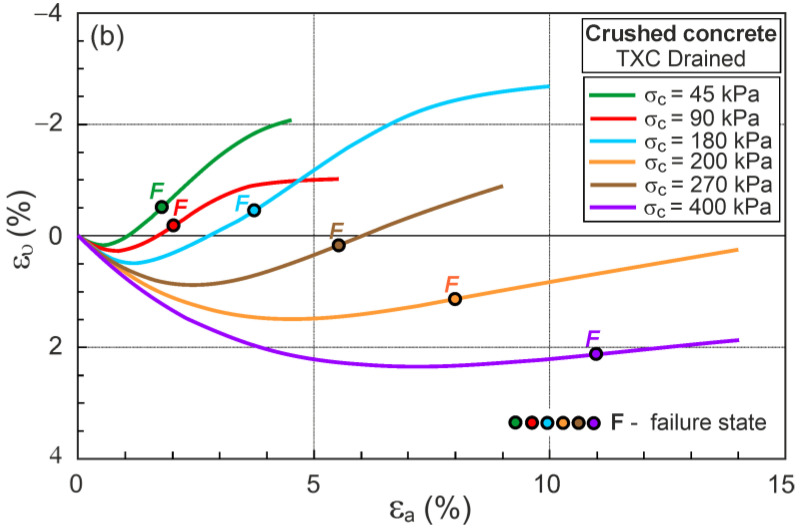
The relationships for drained triaxial compression: (**a**) $q-\varepsilon_{a}$; (**b**) $\varepsilon_{\upsilon }-\varepsilon_{a}$.

The $q-\varepsilon_{a}$ and $\varepsilon_{\upsilon }-\varepsilon_{a}$ relationships for drained triaxial compression, similar to those usually presented in the geotechnical literature, are shown in Figure 12.

The F points representing the DFS’s maximum plastic dilatancy are shown in Figure 12. The locations of F (DFS) points are simply identified for $\eta -D^{p}$ relationships but difficult for $q-\varepsilon_{a}$ and $\varepsilon_{\upsilon }-\varepsilon_{a}$ relationships. The $\eta -D^{p}$ relationships are rarely found in the soil mechanics literature.

### 7.2. Undrained Conditions

**Figure 13:**
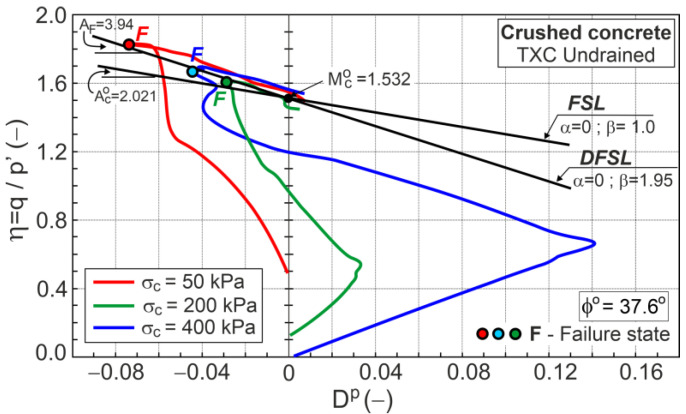
Stress ratio–plastic dilatancy relationship for undrained conditions.

Under undrained conditions, the sample volume does not change during shear (${\delta \varepsilon }_{\upsilon }=0$), and the dilatancy is zero (*D* = 0). The plastic parts of strain increments are equivalent to the elastic strain increments (${\delta \varepsilon }_{\upsilon }^{p}={\delta \varepsilon }_{\upsilon }^{e}$). Assuming that the shear moduli (G) for drained and undrained conditions are equal and the change in Poisson’s ratio (*ν*) during shear is described by Equation (31), the stress ratio–plastic dilatancy can be calculated (Figure 13).

The DFS, represented by F points, is the minimum value of plastic dilatancy ($D_{min}^{p}$), and can be approximated using a straight line (DFSL). The DFSL intersects the vertical axis at $\eta =M_{c}^{o}=1.532$, as in the drained conditions. This means that the critical frictional state angle ($\phi^{o}=37.6{}^{\circ}$) is the same for drained and undrained conditions and does not depend on the stress path. The slope of DFSL in the $\eta -D^{p}$ plane is $A_{F}=3.94$ (Figure 13). For the undrained conditions, $A_{c}^{o}=2.021$ (Equation (18)); hence, $\beta_{F}=A_{F}/A_{c}^{o}=1.95$ is very similar to $\beta_{F}=1.812$ for the drained conditions. This means that the effects of grain crushing on the shear behavior of crushed concrete under drained and undrained conditions are very similar.

**Figure 14:**
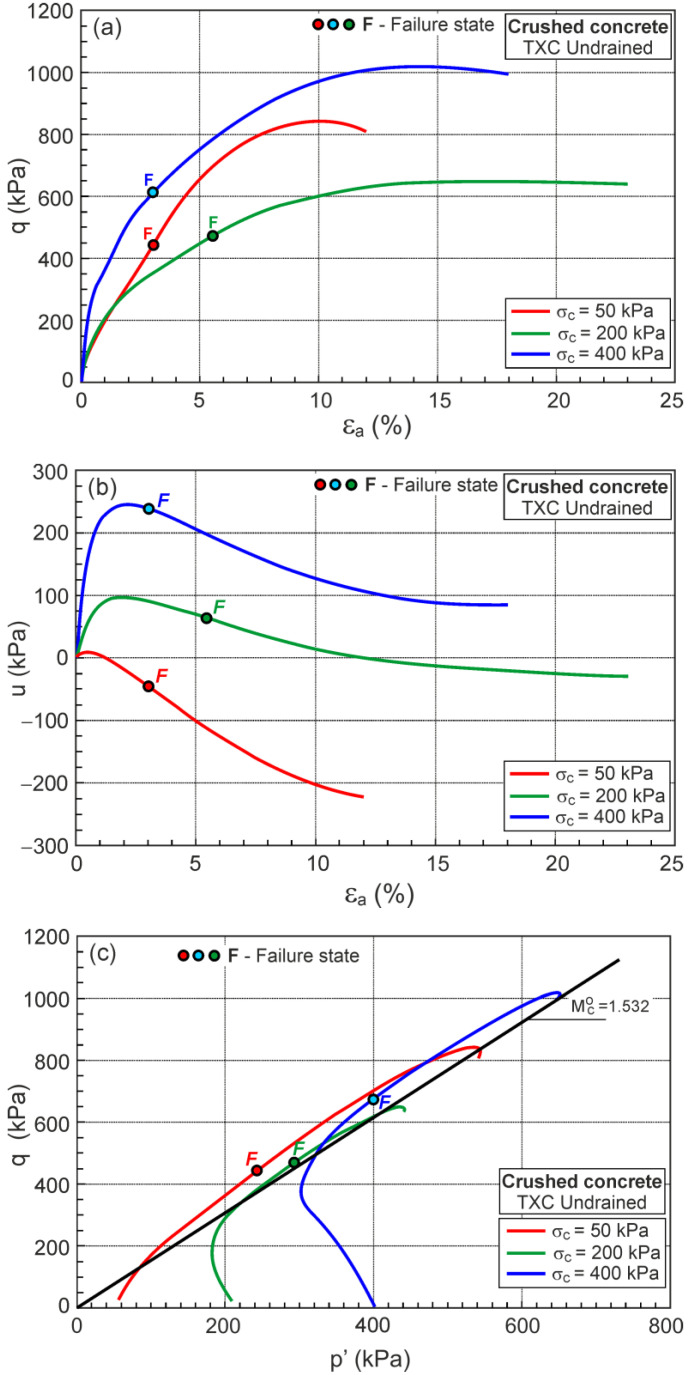
Relationships for undrained triaxial compression tests: (**a**) $q-\varepsilon_{a}$; (**b**) $u-\varepsilon_{a}$; (**c**) $q-p^{\prime }$.

In Figure 14, the relationships $q-\varepsilon_{a}$, $u-\varepsilon_{a}$ and $q-p^{\prime }$ for the undrained triaxial compression tests of crushed concrete are shown.

The F points representing the DFSs are also shown in Figure 14. The location of the F points cannot be simply identified in the $q-\varepsilon_{a}$, $u-\varepsilon_{a}$, and $q-p^{\prime }$ relationships. Therefore, the very important characteristic behavior of soil during undrained shear can be easily identified in the $\eta -D^{p}$ plane.

## 8. Conclusions

The laboratory investigations performed using the triaxial apparatus of the selected geomaterial, namely, crushed concrete, and elastic–plastic modeling using the frictional state concept allowed us to draw some important conclusions. The main conclusions reached from this study are highlighted below.

(1) The characteristic behavior of crushed concrete during triaxial shearing can be described using the frictional state concept.
(2) The dilatant failure state can be easily identified only in the stress ratio–plastic dilatancy plane.
(3) The values of the $\beta_{F}$ parameter, which represents the crushing effect, are almost identical for drained and undrained conditions. This means that the assumptions of the frictional state concept are correct. The crushing effect in the shearing of crushed concrete is observed at low stress levels.
(4) The stress ratio–plastic dilatancy relationship, which is very important in elastic-plastic modeling, can be parametrized using the frictional state concept.

In order to enhance the model’s applicability and expand the practical implications of the research, further improvements can be made to the model considering the variation of a smaller number of parameters. Future research should therefore aim to create a new, even simpler, elastic–plastic model of geomaterials to solve various engineering problems.

## Figures and Tables

**Table 1:** Summary of physical properties of RCA.

Specimen	G_S_ ^a^	d_50_ ^b^	C_u_ ^c^	C_c_^d^	ρ ^e^	ρ _d,max_^f^	ρ _d,min_^g^	e ^h^	OMC ^i^
	−	mm	−	−	g/cm^3^	g/cm^3^	g/cm^3^	−	%
RCA	2.60	2.50	9.09	2.59	1.80	1.710	1.390	0.600	9.5

^a^ Specific gravity; ^b^ Average particle size; ^c^ Uniformity coefficient C_u_ = d_60_/d_10_; ^d^ Curvature coefficient C_c_ = d_30_^2^/(d_60_ × d_10_); ^e^ Bulk density; ^f^ Minimum bulk density of soil skeleton; ^g^ Maximum bulk density of soil skeleton; ^h^ Void ratio; ^i^ Optimum moisture content.

**Table 2:** Summary of chemical properties of RCA.

Specimen	Co	Ni	Cu	Cd	Sulfate	Chlorides	Specific Conductivity	pH
	mg/L	mg/L	mg/L	mg/L	mg/L	mg/L	μS/cm	−
RCA	0.1180	<0.015	0.013	<0.008	112.3	21.6	511.7	8.17
Acceptance criteria *	1	0.5	0.5	0.05	500	1000		

* Official Gazette of the Republic of Poland, Regulation of the Minister of the Environment of 18 November 2014, on the conditions to be met for the introduction of sewage into waters and to land and on substances particularly harmful to the aquatic environment.

**Table 3:** Test conditions characteristics for mixtures used in further analyses.

Test	Type of Test	Compaction State	D_r_^a^	ρ ^b^	ρ_d_^c^	e_0_^d^	p′ ^e^	G_0_^f^
			%	g/cm^3^	g/cm^3^	−	kPa	MPa
RCA-1	CD	dense	65–85	1.751	1.638	0.588	45	75.461
RCA-2	CD			1.772	1.634	0.592	90	98.678
RCA-3	CD			1.793	1.675	0.594	180	177.434

^a^ Relative density of soil; ^b^ Bulk density; ^c^ Dry density of soil; ^d^ Initial void ratio; ^e^ Mean effective stress (shearing); ^f^ Small-strain shear modulus.

**Table 4:** Elasticity parameters of crushed concrete.

$p_{0}$	$e_{0}$	G*	$G_{0}$	a	$a_{\mathrm{avg}}$	K*	ν*
kPa	−	kPa	kPa	−	−	kPa	−
45	0.572	21,215	75,461	3.557	3.758	21,531	0.129
90	0.570	26,229	98,678	3.762		28,849	0.151
180	0.566	44,874	177,439	3.954		43,954	0.119
200	0.491	18,085	−	−		17,714	0.119
270	0.564	31,152	−	−		30,621	0.120
400	0.513	30,868	−	−		24,174	0.052

## Data Availability

The test data presented in this study are available on request from the corresponding author (K.G.) due to their size.
